# Hearing rehabilitation after subtotal cochleoectomy using a new, perimodiolar malleable cochlear implant electrode array: a preliminary report

**DOI:** 10.1007/s00405-020-06098-1

**Published:** 2020-06-05

**Authors:** Stefan K. Plontke, Laura Fröhlich, Sebastian Cozma, Assen Koitschev, Katrin Reimann, Rainer Weiß, Gerrit Götze, Ingmar Seiwerth, Sabrina Kösling, Torsten Rahne

**Affiliations:** 1 Department of Otorhinolaryngology, Head and Neck Surgery, Martin Luther University Halle-Wittenberg, University Medicine Halle, Ernst-Grube-Str. 40, 06120 Halle (Saale), Germany; 2 ENT Department, University of Medicine and Pharmacy “Grigore T. Popa”, Iasi, Romania; 3 Department of Otorhinolaryngology, Klinikum Stuttgart, Olgahospital, Stuttgart, Germany; 4 Department of Otorhinolaryngology, Head and Neck Surgery, University Hospital Marburg, Philipps-Universität Marburg, Marburg, Germany; 5 Department of Radiology, Martin Luther University Halle-Wittenberg, University Medicine Halle, Halle (Saale), Germany

**Keywords:** Acoustic neuroma, Electrode carrier, Intracochlear, Intralabyrinthine, Vestibular schwannoma, Cochlear implant

## Abstract

**Purpose:**

We here report about the first surgical experience and audiological outcome using a new, perimodiolar malleable cochlear implant electrode array for hearing rehabilitation after subtotal cochleoectomy for intralabyrinthine schwannoma (ILS).

**Method:**

Based on a cochlear implant with MRI compatibility of the magnet in the receiver coil up to 3 T, a cochlear implant electrode array was developed that is malleable and can be placed perimodiolar after tumor removal from the cochlea via subtotal cochleoectomy. Malleability was reached by incorporating a nitinol wire into the silicone of the electrode array lateral to the electrode contacts. The custom-made device was implanted in four patients with intracochlear, intravestibulocochlear or transmodiolar schwannomas. Outcome was assessed by evaluating the feasibility of the surgical procedure and by measuring sound field thresholds and word recognition scores.

**Results:**

After complete or partial tumor removal via subtotal cochleoectomy with or without labyrinthectomy, the new, perimodiolar malleable electrode array could successfully be implanted in all four patients. Six months after surgery, the averaged sound field thresholds to pulsed narrowband noise in the four patients were 36, 28, 41, and 35 dB HL, and the word recognitions scores for monosyllables at 65 dB SPL were 65, 80, 70, and 25% (one patient non-German speaking).

**Conclusion:**

The surgical evaluation demonstrated the feasibility of cochlear implantation with the new, perimodiolar malleable electrode array after subtotal cochleoectomy. The audiological results were comparable to those achieved with another commercially available type of perimodiolar electrode array from a different manufacturer applied in patients with ILS.

## Introduction

Hearing rehabilitation with cochlear implants (CI) has shown to be effective even after substantial trauma to the cochlea due to removal of intracochlear schwannomas via subtotal cochleoectomy [1, 6, 7]. During removal of intracochlear tumors, the delicate structures of the modiolus with the spiral ganglion cells in Rosenthal’s canal need to be preserved to enable sufficient stimulation conditions [6]. Thus, there is no safety margin ensuring complete tumor removal. Consequently, tumor growth may result from possible residual tumor cells within the remnants of the spiral osseous lamina and/or the modiolus. In patients with transmodiolar or translabyrinthine tumor growth but a need for hearing rehabilitation, tumor cells will naturally remain in the modiolus and internal auditory canal (IAC) after removal of the intracochlear portion of the tumor. Although these cases are rare, some patients will favor this approach, if hearing rehabilitation has priority for the patient over complete tumor removal or if it is the only remaining chance for hearing rehabilitation if the contralateral side is already deaf [2, 10].

Follow-up with magnetic resonance imaging (MRI) is thus required to assess growth of possible or obligate residual tumor cells. The inner ear and the IAC can be left out from the MRI artifact area of the magnet’s receiver coil if the coil is placed further posterior and superior than in standard CI surgery [13, 15, 17]. In addition, a CI model should be chosen with a high compatibility of the magnet in the receiver coil. Otherwise, complications such as magnet dislocation and pain may occur [3, 4, 14, 15, 18].

A perimodiolar placement with the electrode contacts in close proximity to the spiral ganglion cells has been suggested as one of the factors contributing to the surprisingly good word recognition even after partial or subtotal cochleoectomy for removal of intracochlear schwannoma and CI [6, 20].

Besides electrode array design, advancements in audio processors and speech coding strategies are believed to influence speech and music perception in CI users. Fine structure processing uses the fine structure of a signal to transmit pitch differentiation and temporal cues [11], which has been shown to be advantageous in difficult hearing situations such as speech recognition in noise [19] and listening to music [12].

In this case series, we describe the first experience with a new, perimodiolar malleable electrode in an implant type with known high MRI compatibility and fine structure coding.

## Methods

### Electrode design

**Fig. 1:**
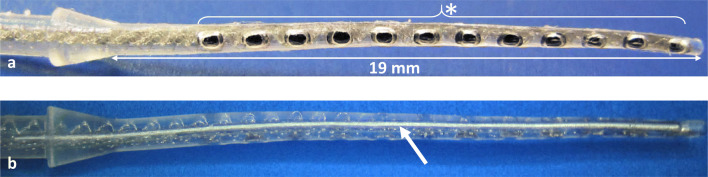
Design of the custom-made electrode array showing the 12 platinum electrode contact pads equally spaced at 1.3 mm intervals at one side (* in **a**) and the shape memory Nitinol wire on the lateral side of the electrode array (→ in **b**). The active stimulations range is 14.3 mm. The diameter at the basal end is 0.8 mm and the diameter at the apical end is 0.5 mm

Based on an existing cochlear implant (CI) model (Synchrony, MED-EL, Innsbruck, Austria), the existing FORM19 electrode array was modified by adding malleable property to be placed around the residual modiolus after tumor removal from the cochlea via subtotal cochleoectomy. In difference to other investigations dealing with shape memory CI electrode arrays, it was not intended to use this property after insertion to optimize the position of electrode contacts but to adapt the electrode array to the cochlear turns before placement in the cochlea. Malleability of the electrode array was achieved by incorporating a wire with shape memory properties (wire length: 25 mm, wire diameter: 0.19 mm) within the silicone elastomer of the electrode array. The 12 platinum electrode contact pads for delivering the electrical stimulation were opened on one side of the array only and the shape memory wire was incorporated in the lateral side of the electrode array still within the silicone elastomer (Fig. 1). The malleable Nitinol wire keeps its shape until 90 °C. Beyond this temperature, the wire will get back to its initial straight configuration. The CI with the malleable electrode array was custom-ordered from MED-EL as a custom-made device (CMD) under the regulations 93/42/EEC, Medical Device Directive.

MRI safety assessment by the manufacturer using comparative power deposition measurements using the ‘Miniature Medical Implant Test System’, revealed that this CMD electrode showed similar power deposition measurements as available standard electrodes under 0.2 T, 1.0 T, 1.5 T and 3 T MRI examination.

### Patients

**Fig. 2:**
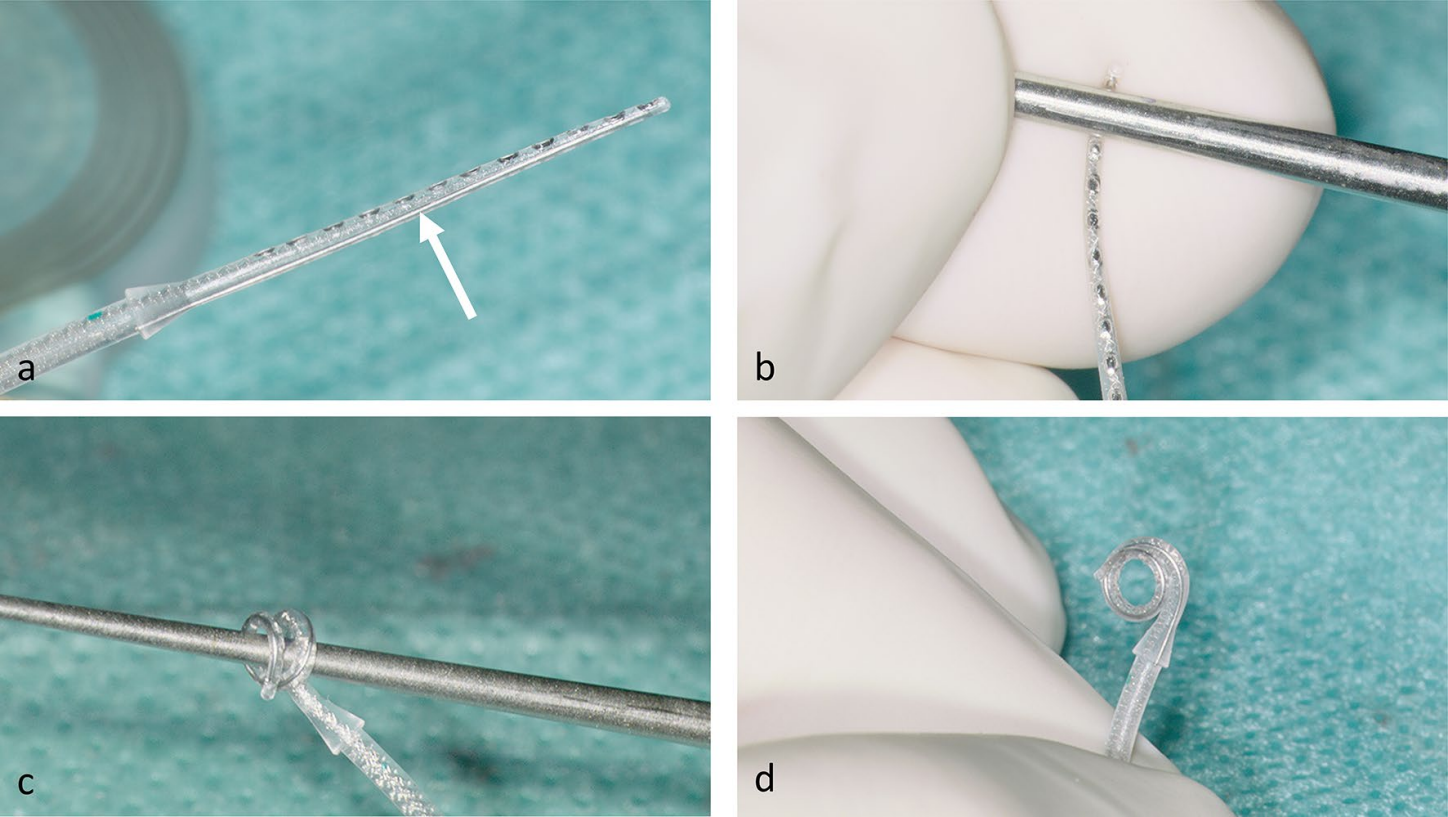
A new, perimodiolar malleable cochlear implant electrode array. The incorporated Nitinol wire (→ in **a**) allowed it to manually shape the electrode array by bending it around the shaft of a conical standard otological instrument like a Rosen needle (**b**–**d**)

**Fig. 3:**
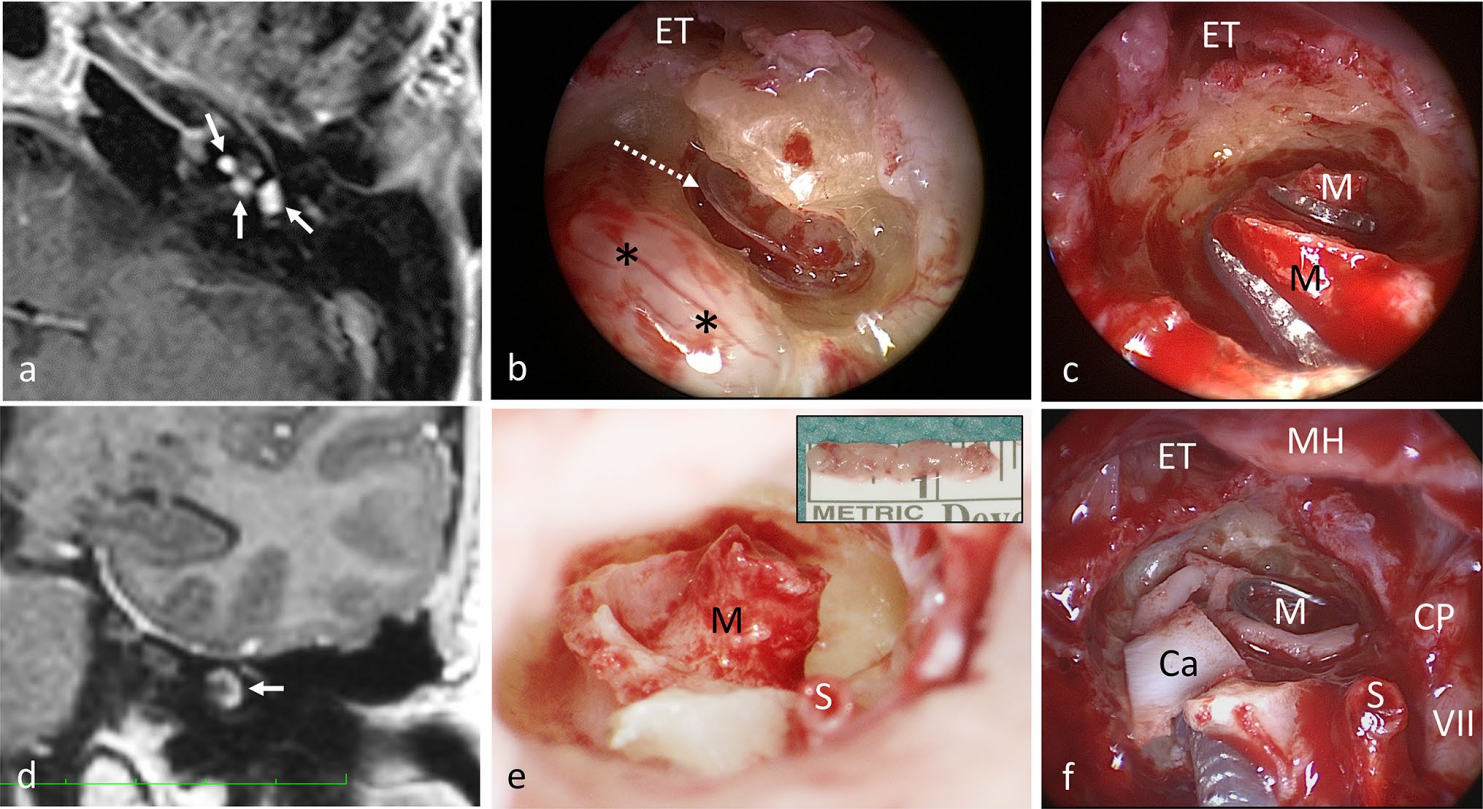
**a** MRI of pat. #1 (T1-w with contrast medium, axial) showing the tumor (→) in the cochlea, in the vestibule and in the fundus of the internal auditory canal. **d** MRI of pat. #2 (T1-w with contrast medium, coronal) demonstrating a solely intracochlear tumor (→) in a young patient. **b, c, e, f** Intraoperative views of patients #1 (**b**, **c**) and #2 (**e**, **f**). The intracochlear tumor parts of patient #1 can be seen in the basal turn (*) while the second turn is tumor free (**b**). The tumor from patient #2 is shown  after removal in the insert in **e**. The perimodiolar formed electrode array was placed around the preserved basal and second turn modiolus (M). **f** Cartilage chips (Ca) were placed peripheral to the electrode array and the defect was closed with a cartilage-perichondrium-island transplant (not shown). Dotted arrow in **b** basilar membrane, *VII* facial nerve, *CP* cochleariform process, *ET* Eustachian tube orifice, *MH* Malleus handle, *S* stapes head, *w* weighted

**Fig. 4:**
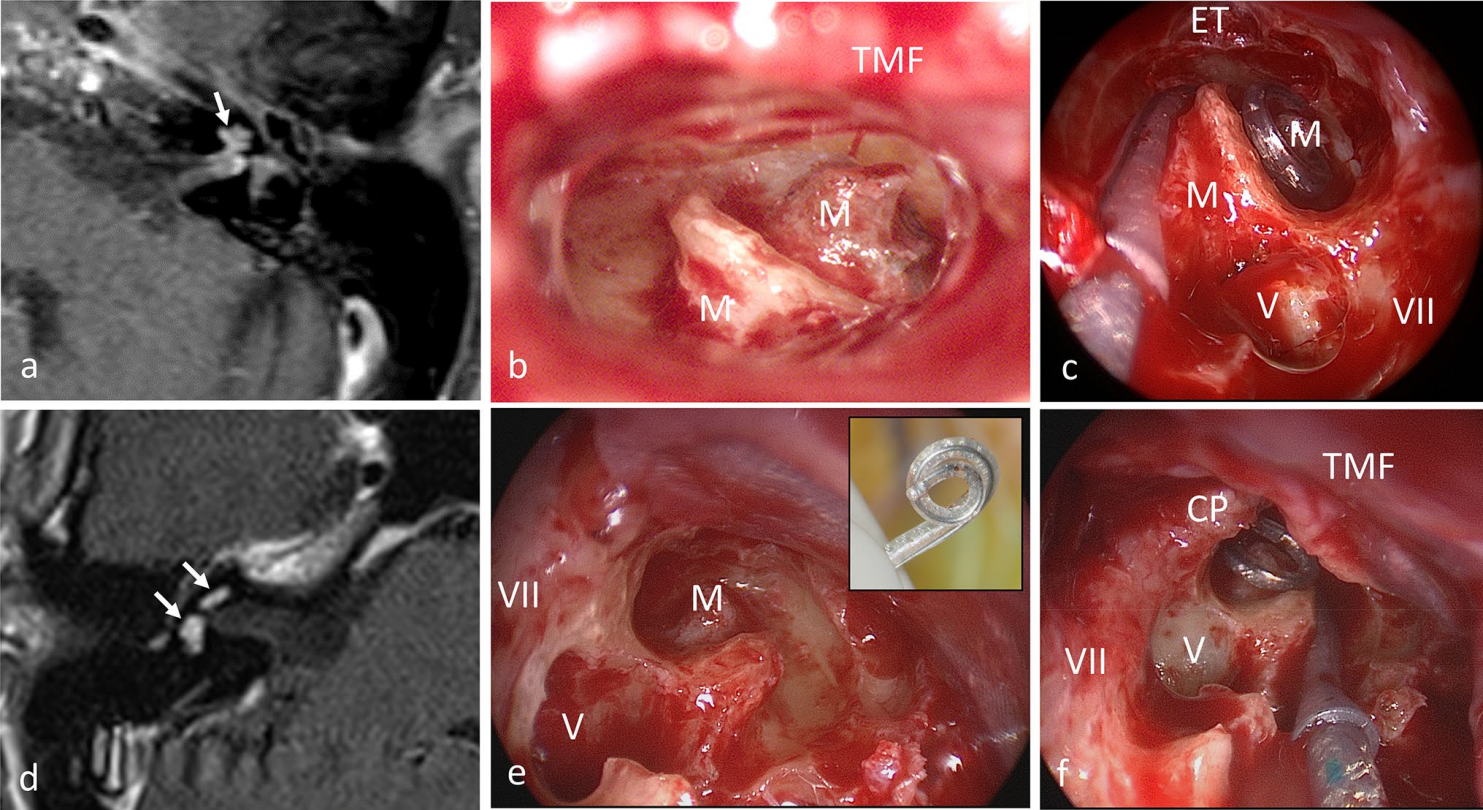
**a** MRI of pat. #3 (T1-w with contrast medium, axial) showing the tumor (→) in the basal and middle turn of the cochlea, and inflammation in the fundus of the internal auditory canal (which disappeared on follow-up MRI). The vestibule contained no tumor but calcified fibrotic tissue (as a result of previous intralabyrinthine bleeding). **d** MRI of pat. #4 (T1-w with contrast medium, axial) demonstrating an intravestibulocochlear tumor (→). **b, c, e, f** Intraoperative views of patients #3 (**b**, **c**) and #4 (**e**, **f**). After tumor removal, the perimodiolar formed electrode array was placed around the preserved basal and second turn modiolus (M) in patient #3 (**b**, **c**) and around the remnants of the modiolus in patient #4 (**e**, **f**). *VII* Facial nerve, *CP* cochleariform process, *ET* Eustachian tube orifice, *TMF* tympanomeatal flap, *V* vestibule, *w* weighted. Insert in **e**: perimodiolarly formed electrode array

Between November 2018 and April 2019, the custom-made device (CMD) was implanted in four patients with intracochlear (1 ×), intravestibulocochlear (1 ×) and transmodiolar (2 ×) schwannomas (Figs. 2, 3a, d and 4a, d). The demographic and baseline audiological data are summarized in Table 1. Patients were extensively informed about the characteristics of cochlear implants and especially the electrode arrays and the receiver coil magnets from the various manufacturers. The four patients in this case series explicitly decided for this custom-made device combining the advantages of a high MRI compatibility and the possibility of a preformed perimodiolar electrode array.

**Table 1:** Demographic data, tumor locations, surgical procedures and pre- and post-surgical audiological data

Nr	Age^a^	m/f	Side R/L	Pre-op hearing (4PTA[dB HL]/ WRS_max_[%]	DOD [years]	Tumor location	Procedure, amount of structural cochlear preservation	Post-op (3 month) WRS_65_ numbers/ monosyllables [%]	Last available post-op WRS_65_ numbers/ monosyllables [%] (months)
1	56	m	L	> 110/0	~ 20	Transmodiolar, intravestibulo-cochlear (basal turn) + Fundus of IAC	Subtotal cochleoectomy + stapedectomy, preservation of basal and 2nd turn modiolus (Fig. 3a–c)	100/45	100/65 (6)
2	30	m	L	71/0	2	Intracochlear (middle turn and apex)	Subtotal cochleoectomy, preservation of basal and 2nd turn modiolus (Fig. 3d–f)	n.a./n.a	90*/80 (6)
3	54	f	L	> 110/0	7	Intracochlear (basal and middle turn)	Subtotal cochleoectomy + labyrinthectomy, preservation of basal and 2nd turn modiolus (Fig. 4a–c)	80/70	100/70 (6)
4	44	f	R	109/0	~ 15	Intravestibulo-cochlear (basal, middle and apical turn)	Subtotal cochleoectomy + labyrinthectomy, preservation of basal turn modiolus (Fig. 4d–f)	80/15	100/40 (12)
Mean ± SD	46 ± 12	2f/2 m	3L/1R	~ 100/0 ± 19/0	~ 11 ± 8			87 ± 12/ 43 ± 28	98 ± 5/64 ± 17

*m/f* male/female, *R/L* right/left, *4PTA* average air conducted pure tone threshold of four frequencies (0.5, 1, 2, 4 kHz), *WRS*_*max*_ maximum number of monosyllabic words understood [in %], *DOD* duration of deafness in the ear with the tumor, *WRS*_*65*_ percentage of words understood (numbers and monosyllables separately tested) in free field at 65 dB SPL with the contralateral ear masked, *CI* cochlear implant, *IAC* internal auditory canal; * romanian bisyllabic/monosyllabic words; *n.a* not available

^a^Age at surgery in years

### Surgery

The surgical procedure for tumor removal through subtotal cochleoectomy and the cochlear defect closure has been described in detail elsewhere [5–7, 9]. A difference to the previously described technique is the missing “round window arch”. Since the electrode is preformed before placement, it cannot be inserted through the extended round window. The CMD electrode array was preformed before insertion by bending it manually with the finger tips and by the help of a “surgical claw” around the conical shaft of a standard otologic instrument (e.g., a Rosen needle) (Fig. 2b–d). The appropriate inner diameter of the electrode spiral was implemented by assessing the dimensions of the modiolus or its remnants with 1.4 and 2 mm suction tips and marking them at the conical shaft of the instrument. Intraoperative microscopic and endoscopic images are shown in Figs. 3 and 4. If other surgical techniques for removing tumor from the cochlear scalae than partial or subtotal cochleoectomy are choosen (e.g., “push-through” or “pull-through”-techniques [1, 7]) or if the electrode is to be inserted into the cochlea without tumor removal (i.e., by pushing a stiff array through the tumor [2]), this CMD electrode array cannot be used.

### Outcome assessment

Outcome was assessed intraoperatively by impedance measurement as well as the recording of electrically evoked compound action potentials (ECAPs) and electrically evoked auditory brainstem responses (EABRs). Postoperatively, the feasibility of the surgical procedure was evaluated by measuring sound field thresholds to pulsed narrow band noise and word recognition thresholds for multisyllabic numbers and monosyllabic words in quiet at 65 dB SPL (WRS_65_) for the German speaking patients and with bisyllabic and monosyllabic Romanian words for one patient (patient #2 in Table 1) from Romania. Free field sound field thresholds and word recognition were measured in quiet with exclusion of the contralateral ear by plugging and masking with white noise.

All patients had signed an informed consent explicitly discussing the specific, custom made nature of the device. This study was approved by the institutional ethical review board (protocol number: 2019-026).

## Results

### Surgical outcome

**Fig. 5:**
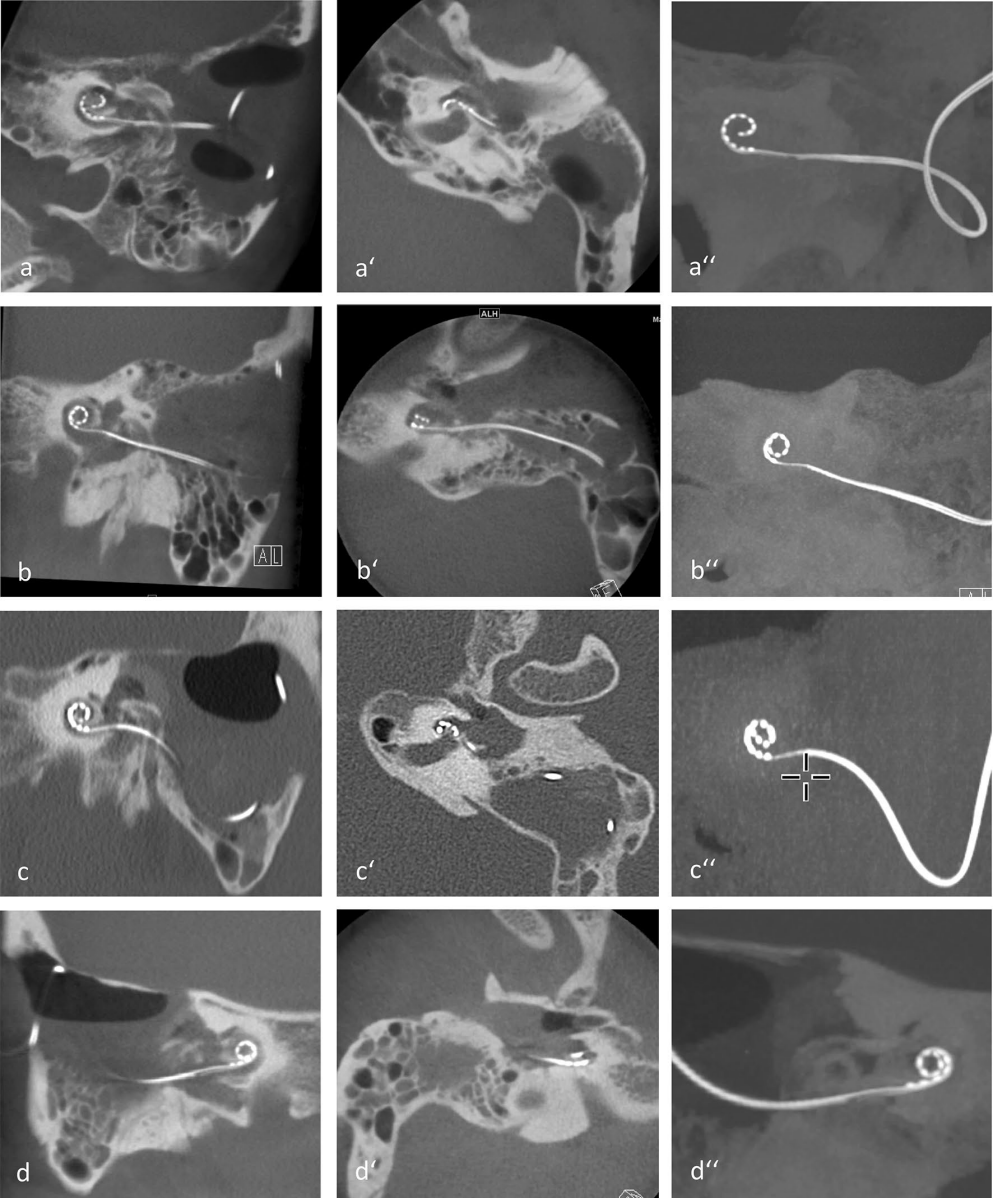
Postoperative Cone Beam Computed Tomography (CT) scans (apart from third row: high-resolution temporal bone CT) showing the electrode arrays in their final position with the remaining parts of the cochlea.  **a–d** Paracoronal multi-planar reconstructions (MPR),  **a**'–**d**' axial MPRs, **a**”–**d**” paracoronal maximum intensity projection (MIP), **a–a**” patient ID 1, **b–b**”  patient ID 2, **c–c**” patient ID 3, **d-d**” patient ID 4 from Table 1

After tumor removal via subtotal cochleoectomy with or without labyrinthectomy, the new perimodiolar malleable electrode array could successfully be implanted in all four patients. Postoperative computed tomography (CT) scans with the electrode arrays in their final position around the remaining parts of the modiolus are shown in Fig. 5. Moderate vertigo for the first 2–4 days after surgery occurred in 3 of the 4 patients (#1, #2, #4). There was one major complication in form of deep venous thrombosis with pulmonary embolism which required therapeutic anticoagulation and a prolonged hospital stay for a total of 14 days.

### Audiological outcome

**Fig. 6:**
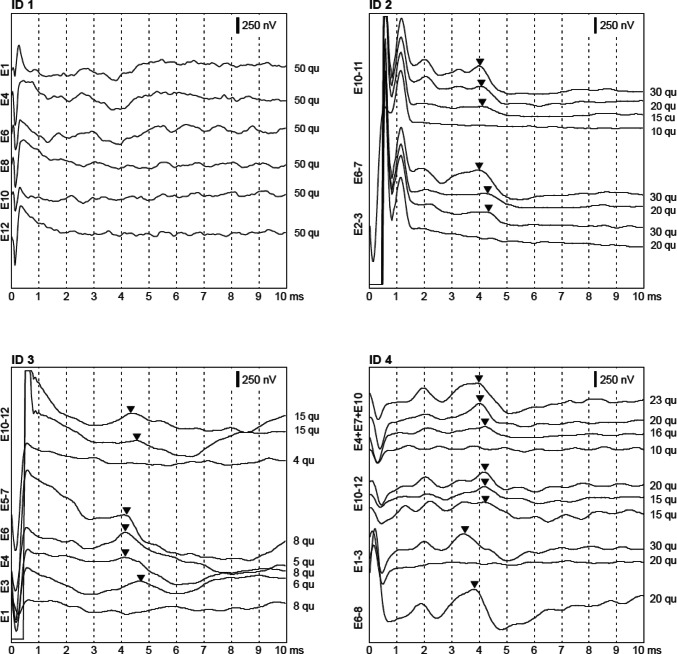
Electrically evoked auditory brainstem responses (EABRs) for all four patients. Stimulated electrodes are marked on the left, stimulation levels at the right sides of the subfigures. Triangles mark the wave V. No EABRs could be recorded in patient #1

**Fig. 7:**
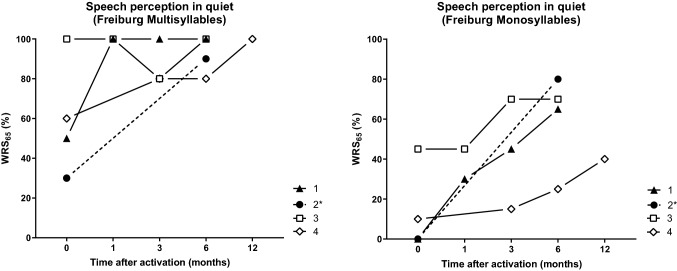
Word recognition score in quiet for multisyllabic numbers (left) and monosyllabic words (right), presented at 65 dB SPL (WRS_65_) as function of the time period after activation of the audio processor. ^*^Romanian bisyllabic and monosyllabic words were used for this patient

Electrophysiological testing during surgery revealed normal impedances below 15 kΩ in all patients. The AutoART algorithm was able to detect ECAP thresholds for four electrodes in patient 1, two electrodes in patients 2 and 3 and one electrode in patient 4. In patients 2, 3, and 4, positive EABRs could be found for simultaneous stimulation of two apical, medial and basal electrodes, respectively. In patient 1, only one electrode was stimulated at a time and no positive EABR could be recorded (Fig. 6). Six months after surgery, the free field pure tone thresholds in the four patients were 36, 28, 41, and 35 dB HL, and the WRS_65_ were 65, 80, 70, and 25% (one patient non-German speaking), (Fig. 7).

### Audio processor fitting and rehabilitation

Audio processor fitting could successfully be performed in all patients. Three patients are using a Sonnet audio processor, one patient is using a Rondo2 audio processor. Postoperatively, the impedances were higher compared to the intraoperative measurement but reached stable values below 15 kΩ after one week.

Maximum comfortable levels (MCL) were found to be between 8.4 qu and 48.2 qu with higher stimulation levels in the apical region in patients 2, 3, and 4 compared to the basal MCL. In patients 2 and 3, the frequency allocation had to be altered based on the patients’ tonotopy with channels 3 and 4 as the lowest frequencies. The most apical electrode 1 was allocated to the fifth frequency band (690–836 Hz) instead.

## Discussion

The surgical evaluation demonstrated the feasibility of cochlear implantation with the new perimodiolar malleable electrode after subtotal cochleoectomy. Considering the substantial trauma through surgical tumor removal from the cochlea, and the long duration of deafness and tumor extensions in some of the patients, the audiological results showed good word recognition which was comparable to that observed with a standard perimodiolar electrode array [6].

Implant integrity could also be confirmed by electrophysiological testing during surgery. Impedances were normal and, while ECAPs were barely present, positive EABR could be elicited in three patients confirming the feasibility of the new electrode. While the recording of EABR in patient 1 was negative, this patient had the highest number of four electrodes with recordable ECAPs. It remains unclear, if the history of deafness for more than 20 years or the stimulation mode during EABR recording with only one electrode stimulated at a time were causing the absence of responses. However, despite the absence of intraoperative EABRs, the postoperative results in patient 1 showed good word recognition scores. All other patients had positive EABRs and showed a continuously increasing speech perception over time after audio processor fitting.

Compared to the previous experience with a preformed electrode array from a different manufacturer (Nucleus CI512, Cochlear ltd., Sydney, Australia) [6, 7], the very tip of the electrode array could not be brought as close to the modiolus, since the wire does not reach to the very tip of the silicon carrier and bending at the very end was difficult. Electrophysiologically, this was also reflected by high stimulation levels needed for electrodes in the apical regions as shown in the patient’s fitting maps and shorter battery life. In two patients, the frequency allocation had to be altered which was not necessary in all but one patient implanted with standard preformed electrode array [6]. It may be speculated that the distance between the apical electrode contacts and the spiral ganglion cells was slightly larger for the CMD electrode array, requiring higher stimulation levels and leading to current spread towards more basal spiral ganglion cells, possibly causing a change of the tonotopy. However, audio processor programming could successfully be performed in all patients without any adverse events.

There are alternative strategies to the subtotal cochleoectomy approach for tumor removal from the cochlea like a “double cochleostomy” with “pull-through” or “push-through” of the tumor [1, 7]. This might result in incomplete tumor removal and increased risk for damaging the subtle structures of the modiolus through insufficient surgical overview. Some authors suggested cochlear implantation without tumor removal by pushing the electrode array through the tumor [2]. However, due to tumor growth, e.g., from the cochlea to the vestibule with increasing symptoms like vertigo [8, 16], this strategy seems only appropriate for selected cases. The alternative strategies, which preserve the cochlear capsule to a higher extend, could not be applied using the CMD described here, since it cannot be inserted through a cochleostomy or an extended round window, respectively. Our surgical technique with a subtotal cochleoectomy for tumor removal, with maximum approximation of the electrode contacts to the spiral ganglion cells in Rosenthal’s canal, and peripheral cartilage placement [5–7], is based on the hypothesis of a reduced spread of the electric field [20]. In our opinion, using a non-perimodiolar electrode with a rather traumatic surgery may result in fibrosis not just peripheral to the electrode array but also between electrode contacts and spiral ganglion cells. However, it may also be possible to “force” a “mid scala” or even a “lateral wall” electrode in to the aspired, close perimodiolar position. In the meantime, various manufacturers offer cochlear implants with “MRI-friendly”, movable magnets in the receiver coil. Thus, the rationale behind choosing the manufacturer and the electrode type reported here, must be relativized.

In addition, in case of a necessary revision surgery, it is not possible to pull out the CMD electrode due to very high risk of “shearing off” the entire modiolus. To date, there is no published experience of revision surgery in cases of intracochlear schwannomas and CI. However, using the technique as described earlier [5–7, 9], simple removal and re-insertion is unlikely after. In these cases, we speculate that the initial surgical procedure needs to be repeated with a possibly higher risk for damaging the modiolus.

## Conclusion

Considering the substantial trauma through surgical tumor removal from the cochlea, the long duration of deafness and the advanced tumor extension in three of the four patients, the preliminary audiological outcomes for this new perimodiolar electrode array showed good results which were comparable to those observed with a standard perimodiolar electrode array, while the MRI-friendly magnet of the receiver coil allows easy imaging follow up.

## Data Availability

All relevant data have been provided in the manuscript. There is no additional or supplementary data.
